# Robotic-Assisted vs. Open Lateral Pancreaticojejunostomy for Chronic Pancreatitis: Perioperative Outcomes and Longitudinal Pain Assessment From a Single-Center Experience

**DOI:** 10.7759/cureus.115025

**Published:** 2026-08-23

**Authors:** Biswabasu Das, Sandeep Kumar Sahu, Durga Bhavani, Neelima Priya

**Affiliations:** 1 Surgical Gastroenterology and Robotic Surgery, CARE Hospitals, Visakhapatnam, IND

**Keywords:** chronic pancreatitis, izbicki pain score, lateral pancreaticojejunostomy, open surgery, perioperative outcomes, postoperative pain, robotic-assisted surgery

## Abstract

Objectives

Chronic pancreatitis (CP) is characterized by debilitating pain, for which lateral pancreaticojejunostomy (LPJ) is an established decompressive procedure in patients with a dilated main pancreatic duct (MPD). This study compared perioperative outcomes and short-term pain-related recovery between robotic-assisted and open LPJ.

Methods

This retrospective analysis of a prospectively maintained database included consecutive adults with CP who underwent LPJ at a tertiary care center in India. Baseline demographic and clinical data were collected preoperatively. Perioperative outcomes were monitored during the index admission and for 30 days following surgery, whereas pain and functional recovery were evaluated preoperatively, at discharge, and three months after the procedure.

Results

The study cohort comprised 23 patients, including 11 who underwent robotic surgery and 12 who underwent the open procedure. Baseline demographic and clinical characteristics were similar between the two groups. All robotic procedures were completed without conversion. The robotic approach was associated with a significantly longer operative duration than the open procedure (332.7 ± 43.4 vs. 166.8 ± 33.1 minutes; p < 0.001). Despite this difference, intraoperative safety and short-term postoperative outcomes were comparable, with neither group experiencing major complications, readmissions, or mortality. Time to oral intake and ICU stay were comparable between groups; however, hospital stay was significantly shorter following robotic LPJ (p < 0.001). While preoperative pain measures were similar, robotic LPJ was associated with significantly lower postoperative pain intensity (30.91 ± 9.44 vs. 54.17 ± 14.43; p < 0.001), reduced work disability (31.82 ± 11.68 vs. 45.83 ± 17.94; p = 0.039), and lower Izbicki pain scores (19.43 ± 4.05 vs. 28.75 ± 6.19; p < 0.001). At three-month follow-up, pain and functional outcomes were low and comparable between groups.

Conclusions

Robotic-assisted LPJ is safe and feasible, with comparable perioperative outcomes and improved early postoperative pain outcomes compared with open surgery.

## Introduction

Chronic pancreatitis (CP) is a progressive, irreversible inflammatory disorder of the pancreas characterized by persistent structural and functional damage. Clinically, it presents with recurrent or persistent abdominal pain, ductal or parenchymal calcifications, endocrine and exocrine insufficiency, and an increased risk of pancreatic malignancy, leading to impaired quality of life (QoL) [1-2]. Pain is the predominant and most debilitating symptom, affecting 80-90% of patients, with nearly one-third ultimately requiring interventional or long-term medical therapy [3-5]. Although CP has a multifactorial etiology, chronic alcohol consumption remains the leading cause worldwide [1].

Surgical intervention is indicated for patients with pain refractory to optimized medical and endoscopic therapy or those with disease-related local complications [6-7]. Approximately 30-75% of patients eventually require surgery, typically in advanced stages of the disease [1-2,4,8]. Surgical options are broadly classified as decompressive procedures, such as lateral pancreaticojejunostomy (LPJ), or resectional procedures, including pancreaticoduodenectomy and distal pancreatectomy. Procedure selection is guided by disease extent, anatomical involvement, and surgical expertise, and the optimal strategy remains debated [9-10]. First described by Puestow and Gillesby in 1958, LPJ is the preferred surgical option for patients with painful CP, a dilated main pancreatic duct (MPD) (>5 mm), and a non-enlarged pancreatic head (≤40 mm) [6,11-14]. LPJ has been shown to provide effective pain relief with low associated morbidity and mortality [2,12]. In selected patients, hybrid procedures, such as the Beger or Frey operations, may be considered and can be performed using open, laparoscopic, or robotic approaches [1].

Technological advances enabled the adoption of minimally invasive LPJ in the early 2000s, with robot-assisted LPJ first reported as feasible in a US case series in 2017 [2]. Compared with open surgery, minimally invasive LPJ aims to reduce wound-related morbidity and analgesic requirements while preserving long-term pain relief and pancreatic function [2,15]. Despite the technical advantages of robotic platforms, evidence specific to robotic LPJ remains limited, with only one study directly comparing robotic and open approaches [9,15]. Accordingly, this study compared perioperative and clinical outcomes between open and robotic-assisted LPJ during implementation of a robotic pancreatic surgery program.

## Materials and methods

Study design and patient population

This was a single-center, retrospective, non-randomized comparative observational study based on clinical data prospectively recorded in the institutional database at Care Hospital, Vizag, India. Consecutive adult patients with CP who underwent LPJ using either an open or robotic-assisted approach between June 2022 and February 2026 were retrospectively reviewed. The two cohorts were defined primarily by distinct time periods, with the open cohort representing the earlier phase (June 2022 to January 2024) and the robotic-assisted cohort the later phase (February 2024 to February 2026). During the latter period, the surgical approach was determined as part of routine clinical practice based on the treating surgeon’s assessment of clinical and anatomical suitability and informed patient preference; no prospectively defined study-specific criteria were applied for allocation to the robotic approach. Only patients with complete perioperative and follow-up data were included. As this was an exploratory retrospective analysis of eligible consecutive cases available during the predefined study period, no formal a priori sample-size calculation was performed. The final sample comprised 23 patients, including 11 who underwent robotic-assisted LPJ and 12 who underwent open LPJ. All 23 patients included in the final analysis completed the three-month follow-up, with no loss to follow-up.

Data collection and ethical compliance

Data collection encompassed patient-level information across the preoperative, intraoperative, and postoperative phases. Preoperative data included baseline demographic variables (age, sex, and body mass index (BMI)), clinical characteristics and comorbidities, biochemical parameters, American Society of Anesthesiologists (ASA) physical status classification, history of pancreatitis-related hospital admissions or prior interventions, and radiological findings such as pancreatic morphology and MPD diameter. Intraoperative data comprised operative duration, type of pancreaticojejunostomy anastomosis performed, intraoperative blood product transfusion requirements, and any intraoperative complications. Postoperative outcomes were recorded during the index hospital admission and up to 30 days following surgery, including pancreatic-specific complications, time to initiation of oral intake, duration of ICU stay, overall length of hospital stay, readmissions, and mortality. Pain and functional outcomes were assessed preoperatively, during the postoperative period prior to discharge, and at three-month follow-up using the Izbicki pain score [16] and its individual components. A standardized postoperative pain-management protocol was followed consistently across both study periods and surgical groups, comprising epidural and intravenous analgesia during early postoperative recovery, followed by oral analgesics until discharge. Given the exploratory nature of this retrospective study and the limited comparative evidence available for robotic LPJ, no single primary endpoint was prespecified. Outcomes were evaluated across two clinically relevant domains: perioperative and short-term postoperative outcomes, and longitudinal pain-related and functional outcomes. Accordingly, statistical comparisons were considered exploratory and hypothesis-generating rather than confirmatory. Ethical oversight for the study was provided by the Institutional Ethics Committee (Approval No. 15/IECC/2026; 1 May 2026). The study adhered to the principles of the Declaration of Helsinki and recognized Good Clinical Practice standards throughout. All participants had previously provided written informed consent for their treatment and for the inclusion of anonymized clinical data in research analyses.

Operative technique

All robotic-assisted and open procedures were performed by the same experienced surgeon, who had already completed the learning curve for robotic surgery, ensuring consistency in operative technique and minimizing inter-operator variability. Robotic cases utilized standardized multiport techniques in accordance with contemporary professional surgical society recommendations. All robotic procedures were performed using the da Vinci X Surgical System (Intuitive Surgical, Sunnyvale, CA). All patients underwent surgery under general anesthesia in the supine split-leg position, which provided appropriate access for the planned operative approach. Pneumoperitoneum was established at Palmer’s point using a Veress needle. Robotic access was achieved by placing three 8-mm trocars in a single transverse row centered on the umbilical level, ensuring an interport distance of approximately 6-8 cm to minimize instrument collision. An additional 5-mm laparoscopic port was placed in the left lateral abdomen. The left lateral robotic port was subsequently upsized to a 12-mm port to facilitate use of the SureForm™ stapler for bowel division and anastomosis. A consistent robotic configuration was maintained throughout the study, using a fenestrated bipolar forceps in the first robotic arm, the optical camera in the second arm, and monopolar scissors in the third. After port placement, the robotic system was approached and docked from the patient's right side. The gastrocolic omentum was divided using an energy device to enter the omental bursa. Anterior retraction of the stomach was achieved with silk hitch sutures anchored to the abdominal wall. The transverse mesocolon was carefully dissected to expose the inferior pancreatic margin, after which the lesser sac was opened in its entirety. The MPD was identified in the body of the pancreas by direct ductotomy using monopolar scissors. When duct localization was challenging, intraoperative ultrasound was employed to facilitate identification. The MPD was laid open longitudinally from head to tail using monopolar and bipolar cautery to ensure adequate drainage. Intraluminal calculi were extracted as identified, and strictures in the body and neck were opened as necessary. Stones within side branches were removed when accessible.

A 30-cm Roux limb was constructed. To improve exposure of the proximal jejunum, the transverse colon was reflected cranially, enabling precise identification of the ligament of Treitz. A mesenteric window was created approximately 20 cm distal to the ligament using bipolar energy and scissors, and the jejunum was divided with a 45-mm blue SureForm™ robotic stapler. To facilitate a tension-free reconstruction, the Roux limb was extensively mobilized and brought into the lesser sac via a transmesocolic opening. Once positioned, it was oriented alongside the pancreatic remnant in preparation for the pancreaticojejunostomy. The jejunum was anchored longitudinally to the pancreatic parenchyma using 3-0 round-body polydioxanone sutures (PDS). A longitudinal enterotomy was created with monopolar electrocautery to match the length of the pancreatic ductotomy. A side-to-side pancreaticojejunostomy was fashioned in a single-layer running manner using 3-0 PDS. Reconstruction commenced with the posterior outer layer, which was sutured from the pancreatic head toward the tail, securing the jejunal seromuscular layer to the pancreatic capsule. The anterior outer layer was subsequently completed in a left-to-right direction while ensuring inversion of the jejunal mucosal edge. A side-to-side jejunojejunostomy was subsequently created. The two jejunal limbs were aligned and anchored with seromuscular sutures. Small enterotomies were made in each loop using monopolar cautery, and a stapled side-to-side anastomosis was performed with a single firing of the 45-mm blue SureForm™ stapler. The common enterotomy was closed in a single-layer continuous inverting fashion using 3-0 V-Loc™ suture. A closed-suction drain was placed adjacent to the pancreaticojejunostomy prior to undocking.

Postoperative management

Following surgery, patients were routinely admitted to the ICU for close postoperative monitoring and, when clinically stable, were transferred to the surgical ward on the first postoperative day. Nasogastric decompression was generally discontinued on postoperative day 2 once gastric output was below 100 mL during the preceding 24 hours. Oral intake was resumed with clear liquids on postoperative day 2 and progressed to a semisolid diet on postoperative day 3 according to individual tolerance. On postoperative day 3, drain fluid amylase levels were measured and abdominal ultrasonography was performed to assess the integrity of the pancreaticojejunostomy and detect any early postoperative collections. Surgical drains were removed on postoperative day 4 when drain amylase concentrations were less than three times the upper limit of normal. Patients with persistently elevated drain amylase levels underwent continued external drainage until resolution. In uncomplicated cases, discharge was typically achieved by postoperative day 5. Octreotide was administered routinely from postoperative days 1 through 3. Postoperative analgesia followed a standardized multimodal protocol consisting of epidural anesthesia and intravenous analgesics during the early recovery phase, followed by oral analgesics until discharge.

Technical considerations: tips, tricks, and pitfalls

Identification of the pancreatic duct could be challenging, particularly in cases with nondilated ducts; intraoperative ultrasound proved valuable in such situations. The jejunal Roux limb was oriented such that its genu lay at the proximal end of the anastomosis adjacent to the pancreatic neck, thereby promoting a tension-free and anatomically favorable alignment. The jejunal enterotomy was intentionally made slightly smaller than the pancreatic ductotomy, as the jejunal opening tended to stretch. Failure to account for this could result in a “dog-ear” deformity at the anastomotic corner, potentially increasing the risk of postoperative pancreatic fistula (POPF).

Statistical analysis

All study data were entered and managed using Microsoft Excel spreadsheets (Microsoft® Corp., Redmond, WA). Descriptive statistics are presented as mean ± SD for continuous variables and as frequencies with percentages for categorical variables. Group comparisons were performed using the chi-square or Fisher's exact test for categorical data and the independent-samples t-test or Mann-Whitney U test for continuous variables, as appropriate based on data distribution. Given the small sample size, multivariable regression or propensity-score-based adjustment was not considered statistically robust. Accordingly, between-group analyses were unadjusted and exploratory, and no causal inference was intended. Statistical significance was defined as a two-sided p-value < 0.05. All analyses were carried out using Stata version 16.0 (StataCorp LLC, College Station, TX).

## Results

Baseline characteristics

The final study cohort comprised 23 patients, including 11 who underwent robotic surgery and 12 who were managed with the conventional open approach. Baseline characteristics are presented descriptively in Table 1.

**Table 1:** Baseline characteristics of the study cohorts Statistical tests used: chi-square or Fisher’s exact test, t-test; significance level: p < 0.05. ASA, American Society of Anesthesiologists; BMI, body mass index; SD, standard deviation

Variables	Robotic (N = 11)	Open (N = 12)	Chi-square value/t-value	p-value
Age, mean ± SD, years	36.55 ± 12.19	47.67 ± 15.33	1.9134	0.0694
BMI, mean ± SD, kg/m^2^	20.23 ± 2.48	19.50 ± 1.88	-0.8028	0.4311
Gender, n (%)				
Male	7 (63.64)	11 (91.67)	2.6504	0.131
Female	4 (36.36)	1 (8.33)	2.6504	0.131
Tobacco use, n (%)	0	0	-	-
Alcohol use, n (%)	0	2 (16.67)	2.0079	0.261
Diabetes, n (%)	2 (18.18)	7 (58.33)	3.8845	0.060
Serum albumin, mean ± SD, g/dL	4.00 ± 0.39	3.74 ± 0.55	-1.2986	0.2081
ASA score, n (%)				
Grade I	7 (63.64)	3 (25.00)	3.4862	0.074
Grade II	4 (36.36)	9 (75.00)	3.4862	0.074
Prior admission for pancreatitis, n (%)	0	3 (25.00)	3.1625	0.124
Prior intervention for pancreatitis, n (%)	2 (18.18)	3 (25.00)	0.1568	0.545
Previous abdominal surgery, n (%)	0	0	-	-
Pancreas atrophy, n (%)				
Normal	1 (9.09)	2 (16.67)	2.6130	0.504
Mild	7 (63.64)	4 (33.33)	2.6130	0.504
Moderate	3 (27.27)	5 (41.67)	2.6130	0.504
Calcified	0	1 (8.33)	2.6130	0.504
Main pancreatic duct diameter, mean ± SD, mm	6.45 ± 5.20	8.33 ± 1.72	1.1842	0.2496

Although no statistically significant between-group differences were identified, clinically relevant imbalances may remain given the small sample size and non-randomized design. Compared with the open cohort, patients undergoing robotic surgery had a lower average age (36.55 ± 12.19 vs. 47.67 ± 15.33 years). Despite this numerical difference, age distribution did not differ significantly between the groups (p = 0.069). BMI, serum albumin levels, and pancreatic duct diameter were similar between groups. A higher proportion of patients undergoing robotic surgery were classified as ASA grade I (63.64% vs. 25.00%), although ASA grade distribution did not differ significantly between the robotic and open cohorts (p = 0.074). Comorbidities, such as diabetes and prior admissions for pancreatitis, were more frequent in the open group, without reaching statistical significance. Overall, baseline variables were well balanced, allowing meaningful comparison of perioperative and postoperative outcomes.

Perioperative and postoperative outcomes

Perioperative and postoperative outcomes of the study cohorts are presented in Table 2.

**Table 2:** Perioperative and postoperative outcomes of the study cohorts Statistical tests used: chi-square or Fisher’s exact test, t-test; *significance level: p < 0.05. DGE: delayed gastric emptying; FFP: fresh frozen plasma; POPF: postoperative pancreatic fistula; PPF: post-pancreatectomy fistula; PRBC: packed red blood cells; SD: standard deviation; SSI: surgical site infection

Variables	Robotic (N = 11)	Open (N = 12)	Chi-square value/t-value	p-value
Conversion to open, n (%)	0	-	-	-
Operative time, mean ± SD, min	332.73 ± 43.38	166.82 ± 33.11	-10.0830	<0.001*
Anastomosis type, n (%)				
Single	0	0	-	-
Double	11 (100.0)	11 (91.67)	-	-
Blood transfusion, mean ± SD, units				
PRBC	1.09 ± 0.30	1.00 ± 0.00	-1.0000	0.3293
FFP	2.00 ± 0.00	1.64 ± 0.81	-1.4907	0.1516
Intraoperative complications, n (%)	0	0	-	-
POPF, n (%)	0	0	-	-
DGE, n (%)	0	0	-	-
PPF, n (%)	0	0	-	-
SSI, n (%)	0	0	-	-
Other complications, n (%)	0	0	-	-
Time to oral intake, mean ± SD, hours	4.73 ± 1.10	3.82 ± 0.98	-2.0412	0.0546
ICU stay, mean ± SD, days	1.82 ± 0.60	2.18 ± 0.39	1.3416	0.1123
Length of hospital stay, mean ± SD, days	6.09 ± 1.08	8.73 ± 0.90	-0.2524	<0.001*
30-day readmission, n (%)	0	0	-	-
30-day complications, n (%)	0	0	-	-
30-day mortality, n (%)	0	0	-	-

No intraoperative conversion from the robotic to the open approach was required, with all robotic procedures completed as planned. Operative time was significantly longer in the robotic group compared with the open group (332.73 ± 43.38 vs. 166.82 ± 33.11 minutes, p < 0.001). Despite longer operative duration, no intraoperative complications were observed in either group; however, the limited sample size precludes definitive comparative conclusions regarding safety. Postoperative outcomes were favorable in both cohorts, with no cases of POPF, delayed gastric emptying (DGE), post-pancreatectomy fistula (PPF), surgical site infection (SSI), or other complications observed. No significant between-group differences were observed in the time to oral intake (p = 0.0546) or length of ICU stay (p = 0.1123). However, the robotic cohort experienced a significantly shorter overall hospital stay (6.09 ± 1.08 vs. 8.73 ± 0.90 days; p < 0.001). There were no 30-day readmissions or mortalities in either group.

Pain and functional outcomes

Preoperative pain scores, including the Izbicki pain score and its individual components, were comparable between the robotic and open groups (Table 3).

**Table 3:** Pain scores at different time points Statistical tests used: t-test; *significance level: p < 0.05. SD: standard deviation

Variables	Robotic (N = 11)	Open (N = 12)	Chi-square value/t-value	p-value
Frequency of pain, mean ± SD				
Preoperative	52.27 ± 23.60	43.75 ± 18.84	-0.9612	0.3474
Postoperative	0.00 ± 0.00	0.00 ± 0.00	-	-
At 3-month follow-up	4.55 ± 10.11	4.17 ± 9.73	-0.0915	0.9279
Intensity of pain, mean ± SD				
Preoperative	48.18 ± 16.01	41.67 ± 16.42	-0.9618	0.3471
Postoperative	30.91 ± 9.44	54.17 ± 14.43	4.5259	<0.001*
At 3-month follow-up	1.82 ± 6.03	2.50 ± 6.21	0.2665	0.7924
Pain medication use, mean ± SD				
Preoperative	3.00 ± 4.10	5.33 ± 5.90	1.0916	0.2874
Postoperative	15.00 ± 0.00	15.00 ± 0.00	-	-
At 3-month follow-up	0.00 ± 0.00	0.08 ± 0.29	0.9555	0.3502
Inability to work, mean ± SD				
Preoperative	31.82 ± 22.61	45.83 ± 20.87	1.5460	0.1371
Postoperative	31.82 ± 11.68	45.83 ± 17.94	2.1968	0.0394*
At 3-month follow-up	2.27 ± 7.54	2.08 ± 7.22	-0.0616	0.9515
Izbicki pain score, mean ± SD				
Preoperative	33.82 ± 10.43	34.15 ± 8.44	0.0831	0.9345
Postoperative	19.43 ± 4.05	28.75 ± 6.19	4.2276	<0.001*
At 3-month follow-up	2.16 ± 3.92	2.21 ± 5.24	0.0253	0.9800

Lower postoperative pain intensity and composite Izbicki pain scores were observed in the robotic cohort, whereas postoperative pain medication-use scores were identical between groups. These findings should be interpreted cautiously given the exploratory analysis and multiple comparisons. Patients in the robotic group reported significantly lower postoperative pain intensity (30.91 ± 9.44 vs. 54.17 ± 14.43; p < 0.001), reduced inability to work (31.82 ± 11.68 vs. 45.83 ± 17.94; p = 0.0394), and a markedly lower postoperative Izbicki pain score (19.43 ± 4.05 vs. 28.75 ± 6.19; p < 0.001). At three-month follow-up, pain scores and functional outcomes were low and comparable between groups, indicating sustained symptom control in both cohorts. Overall, despite a longer operative time, the robotic approach demonstrated excellent procedural safety with no conversions and was associated with significantly improved early postoperative pain and functional recovery, suggesting a clinical advantage over the open approach.

## Discussion

CP is a progressive inflammatory disease characterized by chronic pain, ductal dilation, calcifications, and eventual exocrine and endocrine insufficiency, with alcohol-related and idiopathic causes predominating in our cohort. A significant proportion of patients, particularly those with a dilated MPD, ultimately require surgical intervention. While the optimal surgical approach remains debated, minimally invasive techniques have gained interest. Robotic platforms such as the da Vinci Surgical System facilitate precise pancreaticojejunostomy reconstruction and offer recovery advantages comparable to open surgery. Pain and impaired QoL are the most common indications for surgical intervention in patients with CP [9,17]. The overarching goal of CP management is to improve QoL by alleviating symptoms and arresting disease progression [2,8]. Although pancreatic surgery has traditionally been regarded as one of the most complex surgical disciplines [18], substantial advances over recent decades have improved outcomes; nevertheless, such procedures remain largely confined to centers of excellence [19]. Prior to the advent of robotic platforms, laparoscopic approaches aimed at improving surgical outcomes in pancreatic surgery failed to achieve widespread adoption [20]. Increasing adoption of robotic surgical platforms has broadened the scope of minimally invasive pancreatic surgery, particularly for technically complex procedures [18,21]. By addressing key limitations of conventional laparoscopy, robotic platforms facilitate the execution of technically demanding operations [18,22]. Advantages of robotic surgery include enhanced surgeon control, three-dimensional visualization, improved precision, advanced hemostatic energy devices, and tremor filtration, features that are particularly critical for delicate reconstructive steps such as duct-to-mucosa pancreaticoenteric anastomosis during pancreaticojejunostomy [15,20]. However, robotic surgery is not without limitations, including the absence of tactile feedback, increased costs, longer operative times, bulky equipment, the need for specialized surgical expertise, and challenges related to port placement in multi-quadrant procedures [23,24]. In this context, we present our institutional experience and compare preoperative and postoperative clinical outcomes of robot-assisted LPJ with those of the conventional open approach at a tertiary care center.

Baseline demographic characteristics, including age, BMI, and sex distribution, were similar to those described in earlier reports [1,15,25,26]. Comparison of the robotic and open groups revealed no statistically significant differences in mean age (36.55 ± 12.19 vs. 47.67 ± 15.33 years; p = 0.0694), BMI (20.23 ± 2.48 vs. 19.50 ± 1.88 kg/m²; p = 0.4311), or sex distribution (male: 63.64% vs. 91.67%; female: 36.36% vs. 8.33%). Mean age was lower in the robotic cohort; however, no statistically significant between-group difference was identified. In contrast, a previous comparative study reported significantly younger patients in the robotic LPJ group compared with the open group (39 vs. 54 years; p = 0.035) [15], a finding also supported by reports demonstrating younger age profiles among patients undergoing robotic pancreatic surgery [27]. Comorbidity profiles were comparable between groups. Although a higher proportion of patients in the open group were diabetic (58.33% vs. 18.18%), this difference was not statistically significant. Similar findings have been reported previously, with fewer comorbidities observed among patients undergoing robotic pancreatic surgery compared with open procedures [27]. Prior hospital admissions and interventions for pancreatitis were more frequent in the open group but did not differ significantly between groups. Most patients in the robotic group were classified as ASA grade I, whereas patients in the open group were predominantly ASA grade II; however, this difference was not statistically significant. Tobacco use, alcohol consumption, pancreatic atrophy, and MPD diameter were comparable between groups. These findings are consistent with prior reports demonstrating no significant differences in BMI, sex, duct diameter, or ASA classification between open and robotic LPJ cohorts [15].

Intraoperative and postoperative outcomes are key indicators of surgical safety. Our findings demonstrate that robotic pancreaticojejunostomy remains more time-intensive than the conventional open technique, with operative duration approximately doubling in the robotic cohort (332.73 ± 43.38 vs. 166.82 ± 33.11 minutes; p < 0.001). This observation is consistent with previously published experience [9]. In a prior series involving five patients (two robotic LPJ and three robotic modified Frey’s procedures), the mean docking and console times for robotic LPJ were 22.5 and 345 minutes, respectively [25]. Another case report described an operative time of 388 minutes for robotic LPJ [9], while a separate study reported a median operative time of 210 minutes (range: 191-224), which remained higher than the operative time observed in the open group in our study [26]. Evidence from another comparative series suggests a similar trend toward longer operative times with robotic LPJ (268 vs. 236 minutes). However, unlike the present study, the observed difference did not reach statistical significance (p = 0.412) [15]. Importantly, operative duration in both robotic-assisted and open LPJ is influenced by several procedure- and disease-specific factors. The presence of portal hypertension, smaller pancreatic duct size (which increases technical difficulty), challenges in duct identification, particularly in the absence of intraoperative ultrasound, soft pancreatic parenchyma (associated with increased handling difficulty and bleeding risk), and pancreatic strictures can all prolong operative time irrespective of the surgical approach. These factors should therefore be interpreted as intrinsic contributors to procedural complexity rather than modality-specific limitations. Notably, robotic operative times demonstrate a progressive reduction with increasing surgical experience, reflecting the impact of the learning curve and improved procedural efficiency with greater familiarity in robotic-assisted LPJ. Perioperative blood transfusion requirements were comparable between the robotic and open cohorts, with no statistically significant differences observed in the use of packed red blood cells (PRBC) (1.09 ± 0.30 vs. 1.00 ± 0.00; p = 0.3293) or fresh frozen plasma (FFP) (2.00 ± 0.00 vs. 1.64 ± 0.81; p = 0.1516). These findings are consistent with prior reports in which no patients undergoing robotic LPJ required perioperative transfusion [26].

In the present study, all robotic procedures were completed successfully without conversion to open surgery, consistent with prior reports in which no conversions were required in the robotic group [25]. Notably, no intraoperative adverse events or postoperative complications, including POPF, DGE, PPF, or SSI, were observed in either the robotic or open groups. The perioperative complication profile observed in this study compares favorably with previously published data. A previous comparative study documented perioperative complications in 57% (4/7) of patients undergoing robotic surgery and 68.4% (13/19) of those undergoing the open approach [15]. In the same study, no patients in the robotic cohort developed POPF, whereas two of 19 patients (10.5%) in the open group experienced POPF [15]. Similarly, a case report of robotic LPJ described an uncomplicated postoperative course with no occurrence of POPF [9]. ICU length of stay was similar between the robotic and open groups (1.82 ± 0.60 vs. 2.18 ± 0.39 days; p = 0.1123), whereas the robotic approach was associated with a significantly shorter postoperative hospital stay (6.09 ± 1.08 vs. 8.73 ± 0.90 days; p < 0.001). Likewise, a prior comparative study reported a significantly shorter median length of hospital stay in the robotic LPJ group compared with the open group (5 vs. 7 days; p = 0.009) [15]. Consistent with our findings, another study demonstrated early postoperative recovery after robotic LPJ, reporting a median hospital stay of seven days (interquartile range, 5-7 days) [26]. Additionally, a small series including five patients (two robotic LPJ and three robotic modified Frey’s procedures) documented a mean length of hospital stay of six days in the robotic LPJ cohort [25]. However, as the two cohorts in the present study were treated during different time periods, the observed difference in hospital stay may also reflect temporal changes in perioperative management, discharge practices, or institutional experience and cannot be attributed solely to the surgical approach.

During the 30-day follow-up period, no readmissions, postoperative complications, or mortality were observed in either group. These observations are consistent with previously published comparative data demonstrating similar early postoperative outcomes after robotic and open surgery, including no significant difference in 30-day readmissions and no 90-day mortality in either cohort [15]. Additionally, another study reported no reoperations, zero mortality at both 30 and 90 days, and a low readmission rate, with readmissions occurring only for pain management in three patients (38%) following robotic LPJ [26].

Postoperative pain is a key determinant of early recovery, with inadequate pain control contributing to delayed functional recovery and poorer short-term QoL. QoL represents a patient’s subjective assessment of overall well-being and is conceptually distinct from objective measures of health status [28]. Observational studies have suggested that early surgical intervention in CP may limit disease progression, provide superior pain control, and help preserve pancreatic function [4,29,30]. Supporting this concept, a randomized trial demonstrated that over an 18-month follow-up period, patients undergoing early surgery had significantly lower Izbicki pain scores compared with those managed using an endoscopy-first approach (37 vs. 49; p = 0.02). Supporting the role of timely operative management, one comparative study demonstrated a higher proportion of patients achieving complete or partial pain relief after early surgery than after an endoscopy-first strategy (58% vs. 39%). Although the difference did not reach statistical significance (p = 0.10), the observed trend favors early surgical intervention for long-term pain control in CP [4]. In this study, we evaluated the impact of robotic vs. open surgery on pain-related outcomes in patients with CP. The two groups were well balanced at baseline with regard to pain-related outcomes, including pain frequency, pain intensity, analgesic use, Izbicki pain score, and work disability. Postoperatively, however, patients in the robotic group experienced significantly lower pain intensity (30.91 ± 9.44 vs. 54.17 ± 14.43; p < 0.001), reduced inability to work (31.82 ± 11.68 vs. 45.83 ± 17.94; p = 0.0394), and a markedly lower Izbicki pain score (19.43 ± 4.05 vs. 28.75 ± 6.19; p < 0.001). At three-month follow-up, pain scores and functional outcomes were low and comparable between groups, indicating sustained symptom control in both cohorts. These findings are consistent with prior reports. A small series including five patients (two robotic LPJ and three robotic modified Frey’s procedures) demonstrated complete relief of pain in all patients, with no requirement for oral analgesics at a median follow-up of nine months (IQR, 3-30) [25]. A systematic review reported pain relief rates ranging from 62% to 91% following open LPJ (median 78.5; IQR 23) and from 71% to 100% following minimally invasive LPJ (median 82.5; IQR 12.5), supporting the effectiveness of minimally invasive surgery (MIS) approaches [2]. Importantly, the same review highlighted that MIS is generally associated with less postoperative pain than open surgery, which may translate into shorter hospitalization and faster functional recovery [2]. However, inconsistent reporting of pain outcomes, particularly immediate postoperative pain, was identified as a major limitation in the existing literature, underscoring the clinical relevance of systematic pain assessment following LPJ [2].

Strengths and limitations

Several methodological features enhance the validity of the present findings, including the availability of a prospectively maintained database, adherence to a standardized surgical technique, and implementation of a consistent perioperative care pathway. Notably, the study incorporated a systematic, multidimensional assessment of pain and functional outcomes using the Izbicki pain score and its components at predefined time points (preoperative, immediate postoperative, and three-month follow-up), addressing an important gap in the existing literature where early postoperative pain outcomes are inconsistently reported. Nevertheless, the findings should be interpreted in light of the study's retrospective, non-randomized, single-center design, which may introduce selection bias and limit the generalizability of the results. The relatively small sample size limits the ability to detect uncommon complications, readmissions, or mortality; therefore, the absence of these events should not be interpreted as evidence of equivalent safety. As an exploratory retrospective study without a prespecified primary endpoint and involving multiple comparisons, the findings should be considered hypothesis-generating. The non-randomized treatment allocation and unmeasured factors, including patient preference and socioeconomic considerations, may have introduced selection bias. In addition, postoperative pain assessment may have been influenced by assessment timing, ongoing analgesic use, and the non-blinded observational design, warranting cautious interpretation of early pain-related differences. The short follow-up precluded assessment of long-term outcomes. Study-specific economic data, including operative, hospitalization, and total treatment costs, were not available; therefore, cost-effectiveness could not be evaluated. Robotic LPJ also requires longer operative times and specialized expertise. Larger prospective, multicenter studies with prespecified endpoints, longer follow-up, and appropriate economic evaluation are warranted to validate these preliminary findings.

## Conclusions

Robotic-assisted LPJ appears feasible in appropriately selected patients with CP, with no conversions or major perioperative safety events observed in this limited cohort. Although shorter hospital stay and favorable early postoperative pain and functional outcomes were observed compared with the open cohort, these findings should be considered preliminary and hypothesis-generating rather than evidence of superiority. Larger prospective, multicenter studies with prespecified endpoints and longer follow-up are required to confirm these observations and better define the role of robotic LPJ in clinical practice.
